# An Essay on Lessening Pain and Facilitating Difficult Parturition

**Published:** 1820-09-01

**Authors:** 


*An Essay on the Means of lessening Pain and facilitating certain Cases of difficult Parturition.* By WILLIAM P. DEWEES, M. D. Philadelphia. 8vo. pp. 156.*

IT is with much satisfaction that we notice this Essay. Its subject is so important and so interesting to humanity, that, in bestowing more than an ordinary share of attention upon its contents, we hope to perform a service, by no means unacceptable to our readers.

The author, whose experience has been very extensive, and whose character as a successful and scientific accoucheur ranks very high, is entitled to great attention; he writes like a practical man, and, therefore, writes usefully. Sensible of the very great deference which is due to the experience and opinions of Dr. Dewees, we will not pretend to enter into a critical examination of his doctrines and practice, but proceed to give an analytical account of his Essay.

After having described the anatomy of the uterus, the author proceeds to the physiological consideration of this organ, in relation to its functions in the process of parturition. The views which he takes on this head are original, and he supports them with much ingenuity of argument.

The uterus has always been considered as one individual organ, with regard to the functions of its different parts; that is, the body, neck and fundus of this organ, are held as a single viscus, whose actions are similar and dependent on each other. To this opinion Dr. Dewees objects, he observes,

---

* This being a strictly Analytical Review, we have inserted it nearly verbatim, from the first volume of the AMERICAN MEDICAL RECORDER. *Editor.*

"I cannot help regarding the neck of the uterus as a distinct and independent part from the body and fundus, and as having its own peculiar laws and actions; and that this separation of powers is absolutely necessary to the explanation of some of the phenomena exhibited by health and disease, and the influence of certain agents on these parts." 193.

His reasons for thinking so are,

1. The fundus and body may be greatly distended, without affecting the condition of the neck; thus, during the first six months of pregnancy, the former parts are generally distended, whilst the latter part remains nearly unaltered.

2. After the sixth or seventh month, the neck begins to unfold, whilst the other parts remain nearly stationary.

3. The neck may be affected by disease, while the fundus and body remain free; and *vice versa*.

4. The different conditions in which these parts of the uterus are at the same time. In labour, the office of the body and fundus is diametrically opposite to that of the neck; whilst the former contract, the latter expands.

The author does not accord with those who believe that the mouth of the uterus, in a natural and favourable labour, is dilated by the mechanical power of the ovum, forcing it asunder, as it were, by a wedge. His reasons for dissenting from the commonly received opinion on this head, appear to us very ingenious and altogether valid. As it appears evident, that the *circular* must be weaker than the *longitudinal* fibres of the uterus, the dilatation of the mouth of this organ, during the first stage of labour, is considered by the author, as the necessary result of the contraction of this latter set of fibres, which alone seem to be concerned in the propulsion of the fœtus.

"By the contraction (says he) of the longitudinal fibres the length of the uterus diminishes; this puts the circular fibres upon the stretch, since the uterus cannot diminish in one direction, while the mouth of the uterus remains shut, without augmenting in another; therefore the circular fibres are a little distracted, and they immediately co-operate with the longitudinal, and force the uterus, with its contents, lower into the pelvis. This kind of action is reciprocated for some time; but the circular fibres eventually yield to the influence of the longitudinal; first, from their having expended a portion of their power in maintaining a state of contraction so long; and, secondly, from their being absolutely the weaker fibres. Hence the circular fibres of the neck relax; and hence the dilatation of the mouth of the uterus." 194.

Under the head "of the contraction of the fundus and body of the uterus," the author advances the following positions.

1. "The contraction of the circular fibres is not attended with pain.

2. "Their contraction, however violent, does not forward the child.

3. "They do not possess the power of alternate contraction in the same degree as the longitudinal fibres; and that they may exert this power, it is necessary, at first, to have them distracted by some force or other.

4. "The pain in labour depends, in a great measure, if not entirely, upon the contraction of the longitudinal fibres.

5. "The changes which the uterus has suffered from civilization and refinement must be chiefly confined to its longitudinal fibres." P. 195.

The arguments offered in support of these positions are both ingenious and forcible. There is also much ingenuity and appearance of correctness in our author's mode of accounting for those changes which refinement and civilization have produced in the female constitution, and which renders parturition more painful and tedious than it is with the women of savage nations. He says,

"From what has been said, it appears that the pain attending uterine contractions, depends upon certain physical changes, which the longitudinal fibres have undergone from the cause just mentioned (i. e. civilization and refinement). Why a particular set or given direction of fibres should have suffered more than another may be impossible to determine; but that they have, we believe to be most certain. This change, however, is by no means confined to the uterus, as every straight muscle of the body appears to have participated with it, since it is admitted that the man of the civilized world has lost much of his original strength. On the other hand, the circular muscles, as far as we can determine, have lost nothing of their primitive power; since it is more than probable, that the various sphincters, among which may be reckoned the circular fibres of the mouth of the uterus, perform their duty as effectually, and as powerfully, as in the time of our first parents." 195.

The cause of pain and difficulty of labour, consisting for the most part in a certain condition of the soft parts immediately concerned in labour, and especially *in an unnatural rigidity of the mouth of the uterus,* is next considered under the following heads.

1. When rigidity arises from the circular fibres maintaining their contraction too long; but unattended with inflammation.

2. Rigidity attended with inflammation.

3. Rigidity arising from previous local injury.

4. Relative rigidity, proceeding from disproportionate powers between the longitudinal and circular fibres.

5. Tonic rigidity, where the circular fibres remote from the mouth embrace the child too powerfully.

Rigidity of the first kind is subdivided into three varieties: A. Where the subject is very young, arising, as the author supposes, from the uterus not having yet had its complete state of developement when impregnation took place; though sufficiently for the purpose of gestation. B. Where the subject is not very young. The parts concerned in parturition, not having been employed early, according to the design of nature, seem to have forgot a part of their duty. In this variety much benefit may be derived from the use of an antiphlogistic diet, keeping the bowels freely opened, and occasionally losing blood some time before the period of gestation. C. Where the action of the uterus is prematurely excited.—As it must be always useful to be able to distinguish this variety from the two last mentioned, the following remarks are mentioned as, for the most part, indicating this variety. 1. When the uterus is prematurely excited into action, (as at the eighth month) we can sometimes feel the os tincæ. 2. When the mouth of the uterus is found rigid, both in the absence and presence of pain. 3. When the membranes, touched through the mouth of the uterus, are found less tense than when the uterus is naturally disposed to labour. 4. When the pains are more irregular in their succession and continuance. 5. When "there is no secretion of mucus, nor disposition in the perinæum to relax." 6. When "there is no immediate subsiding of the abdominal tumours."

*Rigidity with Inflammation.* The three varieties mentioned above, are all liable to inflammation: 1st. From local irritation either of the presenting part acting mechanically on the mouth of the uterus, or from the improper interference of the midwife. 2. From improper diet or drink.

"When inflammation comes on, the woman becomes extremely restless, and does not enjoy the calm which is common at the cessation of pain; the vagina becomes hot and dry; the mouth of the uterus thickens and becomes more yielding; the secretion of mucus, if it had taken place, ceases; the pulse becomes quick, frequent and hard; the respiration hurried; the head much pained; the face flushed; great thirst; the skin hot and dry, or profusely sweating." P. 196.

*Relative Rigidity.* This may happen from a variety of causes, but we shall only notice one; namely, a kind of apoplexy of the uterus.

"This is known by labour having come on kindly at first, and gradually diminishing in force; by the mouth of the uterus having a disposition to dilate; by its thickening; by the presenting part not protruding during pain; by the pain extending itself all over the abdomen; by the woman's complaining of a sense of suffocation; by a hard and full, or depressed or labouring pulse; by the irregularity of the pains both in force and frequency." 197.

*Tonic Rigidity.* "This only occurs where the waters have drained off a long time, and the whole of the internal surface of the uterus is closely applied to the body of the child."

After having spoken of the various causes of *rigidity*, the author proceeds to "say a few words on the principal remedies which have been employed with a view to relieve it."

*Opium.* This still continues a favourite remedy with most accoucheurs, for the rigidity of the os uteri. The author objects strenuously to its employment in such cases. He says,

"I have often tried it myself, and have often seen it employed by others, without, in a single instance, producing the effect for which it was prescribed; sometimes it evidently did harm. It has, however, undoubtedly been used with advantage in those cases where the uterus had been prematurely excited into action; it has suspended the contractions until the proper time; and when they were renewed, the uterus was healthily disposed, and the labour soon finished. But here it was given not to dilate the mouth of the uterus, but to suspend the contractions of the longitudinal fibres. Nor can this article be considered as an innocent one; we believe it to be extremely mischievous, in many cases converting the rigidity without inflammation or fever, into those with them. This I have more than once seen, and but too frequently had reason to regret." 197.

*Warm Bath.* This remedy is not much to be depended upon. The author says, "the result of my experience, inquiries, and observations, on this point, may be reduced to three heads:"

1. It is almost always inconvenient.
2. It is sometimes ineligible.
3. It is always limited, and uncertain in its effects.

*Blood-letting.* "This remedy (says the author) is by no means a new one in labour; but employed for the express purpose of diminishing pain, and subduing the various species of rigidity just spoken of, and carried to an extent that will ensure these objects, that is, diminishing pain, disposing the os uteri to dilate, the external parts to unfold, and cicatrices to yield, originated, as far as I know, with myself." "We can (he continues) recommend with a confidence, that should only be produced by experience, this operation, not only as a safe, but a certain remedy for all the objects we have just mentioned. This remedy was at first suggested to me by accident. In the summer of 1789, I settled at Abington, and was quickly introduced to a large share of obstetrical practice; in September of that year, my attendance was bespoke for Mrs. W—, whom I was informed had suffered every thing but death, from her labours; the crotchet had several times been employed to effect the delivery of her children. She looked forward with great solicitude and apprehension; and, indeed, almost considering herself a certain victim to the approaching labour. I had also very great fears for my patient, as I was young, and had not much experience; these forebodings were very much augmented, by my being called to her under a severe hæmorrhage from the lungs, which quickly reduced her to a state of extreme debility. Before she recovered from this state of weakness, she was taken suddenly in labour, which increased my apprehensions almost to despair, lest she should die under my hands. As I approached the house, I was met by several of her friends, who, with great earnestness, begged me for *God's* sake to make all possible haste; I proceeded immediately to her bed-side, and in about fifteen minutes delivered her of a fine healthy child. No accident supervened." 198.

Thus receiving an important hint from Nature, he resolved to imitate this example in the first case that should occur to him, where delivery was rendered tedious or painful, by rigidity of the parts. He very soon met with an opportunity of putting this new plan of treatment into practice; and the result was highly satisfactory.

Twenty-three cases are related by the author, in which copious and prompt bleeding was employed with the most happy effect. We cannot give a more correct and satisfactory view of this practice, than by transcribing a few of these cases.

"*Case* 3. June 11th, 1792. Mrs. F——, aged seventeen, very small of her age, never menstruated until after marriage; was taken in labour with her first child; pain came on very gradually for the first few hours, then augmented very considerably for some time, and then subsided almost altogether; this flagging of the pains was considered as a proof of weakness, and to obviate it, stimulating drinks were liberally given; pepper, thyme, ginger, and onion-tea, had each their trial, without advancing the labour. Her friends became alarmed, and I was sent for; I found her with much fever, severe pains, profuse sweats, hot vagina, swelled labia, and rigid os tincæ. I proposed to bleed her, but this she would not permit; she was placed in the warm bath by way of substitute; mild drinks were given, and her bowels were opened by injection. Warm water was frequently thrown up the vagina, but without any observable effect; I again proposed the bleeding, but it was again rejected. As I had observed that bleeding had done good almost in proportion to the sickness it excited, I thought of giving emetic tartar in small doses, until nausea was produced; I soon brought the stomach to this state, which was kept up with considerable severity for two hours, but without any good effect. I now urged the bleeding as the only chance of benefiting her; to this, at length, she reluctantly submitted. She was bled twice in an hour, the last of which was copious, and had the long looked-for effect; the uterus dilated almost instantly after the bleeding, and the external parts yielded without any difficulty: the child was delivered in half an hour."

"*Var.* 2. *Case* 4. August 30, 1790. M. M. in labour with her third child; she had suffered very severe pains for thirty six hours; the waters had been evacuated twelve hours; the vagina hot and dry; the external parts much swoln; the mouth of the uterus thick, firm, and but little dilated; much fever; bounding pulse; severe head-ache; great thirst; much anxiety and restlessness. I bled to about fifteen ounces, but with no evident advantage. At the end of an hour she was bled twenty ounces more; this seemed to affect her considerably, but its use was transient. She was presently bled twenty ounces more; she became extremely sick, the parts quickly dilated, and she was delivered in half an hour more."

"*Case* 14. February 13th, 1805. Mrs. C——, with her first child; she had been forty-eight hours in labour when I was called; the waters had discharged fourteen hours; her pains severe, but irregular; the mouth of the uterus open to about the size of a quarter of a dollar, but very rigid; the vagina, &c. very hot and tender; pulse frequent and hard; she supposed she had just entered her eighth month, and was seized with pains in consequence of a fall; a midwife was sent for, and she endeavoured, by stimulating drinks, frequent and rude touching, to provoke labour. She was bled twice in four hours, to the amount of twenty-two ounces; received a purgative injection, which operated well, but without producing any change in the uterus. The head presented naturally. Two hours more were allowed to pass, with a hope of things doing better—but no alteration being produced, I made Mr. King (a young gentleman who staid, at my request, with the patient) tie up her arm while standing on her feet, and take blood until she nearly fainted; she was then laid in the bed, and after an exemption from pain for about fifteen minutes, they came on very rapidly; the mouth of the uterus was found completely dilated, and the child was delivered in a quarter of an hour more." 200.

Having stated a variety of cases of difficult and painful labour from rigidity, our author goes on to speak of difficulty of labour, arising from want of force in the uterus. In such cases, Dr. Dewees considers the ergot a very valuable remedy. "It would appear, (he says) from all I have been able to collect, and from all I have observed, that it rarely fails or disappoints, when properly prescribed."

It appears, by a paragraph quoted from the Dict. Rais. Univers. d'Hist. Natur. of Bomare, that the ergot was in common use before the year 1774, "and was prescribed for the very cases, for which it is at present given; and that the effects noticed after its exhibition, were as prompt as they are now found to be."

"This remedy (our author remarks) is regarded as a stimulant of no mean power; but I must confess, I have never witnessed any direct operation upon the sanguiferous system. I have therefore, of late, paid little attention to the state of the system, when about to exhibit it. Its operation appears to be very evanescent, and to be exclusively confined to the muscular fibres of the uterus. Indeed, it appears to be one of those rare substances, which are justly entitled to the name of specifics."

The situations in which the exhibition of this article becomes proper, are,

1. Where, from long and violent efforts to overcome rigidity, the contractions of the uterus become feeble, and, though the soft parts may at last be relaxed, are insufficient to propel the fœtus.

2. Where the efforts of the uterus, though slow, and not very powerful; yet, if sufficiently long continued, may effect the delivery, and the labour be complicated by any accident which would render its speedy termination desirable

3. Where the head of the child has been separated from its body, and left within the uterus.

4. "Where the placenta has been prevented from being thrown off."

5. In dysmenorrhœa.

We have thus given a full account of this excellent Essay. It is written in a perspicuous and unaffected style. The subject is well arranged, the reasoning ingenious and logical, and the matter well condensed.

INSANITY.

